# Regulation of Human Adenovirus Alternative RNA Splicing by the Adenoviral L4-33K and L4-22K Proteins

**DOI:** 10.3390/ijms16022893

**Published:** 2015-01-28

**Authors:** Roberta Biasiotto, Göran Akusjärvi

**Affiliations:** Department of Medical Biochemistry and Microbiology, Science for Life Laboratory Uppsala University, Uppsala 75123, Sweden; E-Mail: roberta.biasiotto@imbim.uu.se

**Keywords:** adenovirus, L4-33K, L4-22K, RNA splicing, SR proteins, PKA, DNA-PK

## Abstract

Adenovirus makes extensive use of alternative RNA splicing to produce a complex set of spliced viral mRNAs. Studies aimed at characterizing the interactions between the virus and the host cell RNA splicing machinery have identified three viral proteins of special significance for the control of late viral gene expression: L4-33K, L4-22K, and E4-ORF4. L4-33K is a viral alternative RNA splicing factor that controls L1 alternative splicing via an interaction with the cellular protein kinases Protein Kinase A (PKA) and DNA-dependent protein kinase (DNA-PK). L4-22K is a viral transcription factor that also has been implicated in the splicing of a subset of late viral mRNAs. E4-ORF4 is a viral protein that binds the cellular protein phosphatase IIA (PP2A) and controls Serine/Arginine (SR)-rich protein activity by inducing SR protein dephosphorylation. The L4-33K, and most likely also the L4-22K protein, are highly phosphorylated* in vivo*. Here we will review the function of these viral proteins in the post-transcriptional control of adenoviral gene expression and further discuss the significance of potential protein kinases phosphorylating the L4-33K and/or L4-22K proteins.

## 1. Introduction

During a few hectic months in the spring of 1977 the laboratories of Rich Roberts and Phil Sharps made the startling discovery that adenovirus genes were encoded at discontinuous genomic positions [1,2]. Both groups used electron microscopy to map the location of individual adenovirus mRNAs on the viral chromosome. They observed that the mRNAs expressed from the adenoviral major late transcription unit (MLTU) had unpaired tails both at the 5' and 3' end of the mRNA. The tail at the 3' end was expected since that represented the poly(A) tail, which is added post-transcriptionally to a mRNA. However, the tail at the 5' end was completely unexpected. Subsequent studies showed that it was composed of three short segments (exons) derived from discontinuous places on the viral genome and separated by intervening sequences (introns) that were excised during the maturation of the mRNA. The results were first presented to a larger scientific audience at the Cold Spring Harbor Symposium on “Chromatin” in May 1977. The description of the split gene concept represented a major turning point in scientific thinking. Thus, the exon-intron arrangement of genes was rapidly shown to be the rule rather than a virus-specific quirk. For many scientists working in various model systems it must have had hard to explain results that immediately became understandable once the radical split gene model was presented because at the time when the symposium volume was finally printed several groups had been able to supplement their chapter with data showing the existence of exons and introns also in their experimental systems.

**Figure 1:**
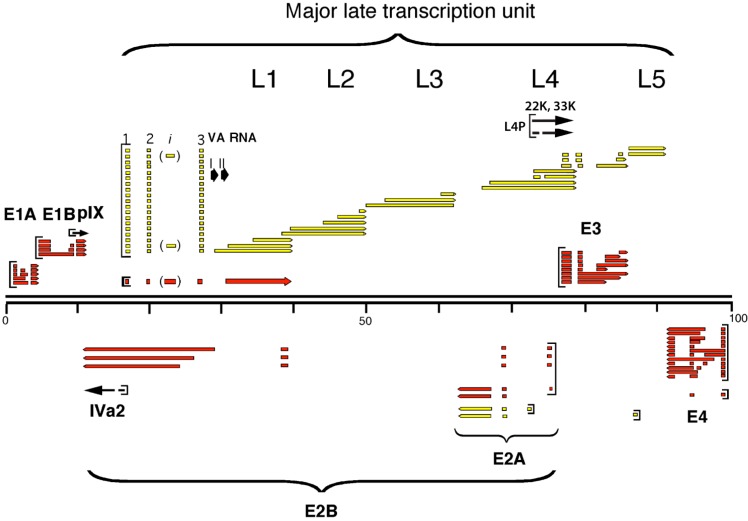
Schematic representation of the Ad5 genome and the expressed viral transcripts. The early transcripts are shown in red and the late ones are marked in yellow. Intermediate genes are indicated as black arrows.

The mechanism and regulation of RNA splicing are described in detail in other chapters in this special issue of the International Journal of Molecular Sciences. Most adenovirus transcription units encode for a complex set of alternatively spliced mRNAs (Figure 1). Here we will review the current knowledge of how adenovirus major late pre-mRNA splicing is regulated, with a specific emphasis on the function of the viral L4-33K and L4-22K proteins. For a description of *cis*-acting elements and trans-acting factors controlling adenovirus E1A and E3 alternative splicing we refer to other reviews [3,4].

## 2. Adenovirus Alternative RNA Splicing

Adenoviruses are small non-enveloped linear double stranded DNA (dsDNA) viruses encoding for about 30–40 genes, whose products interfere with many of the biosynthetic machineries in the infected cell. Adenoviruses are particularly interesting model systems to study because of their clinical potential as a viral vector for cancer gene therapy [5] and because of their association with the pathogenesis of several acute and chronic diseases [6]. To streamline the replication cycle the virus takes control of many of the biosynthetic processes in the cell, like alternative RNA splicing and polyadenylation in order to expand the coding potential of the limited viral genome. Of specific interest for this review is the observation that the production and accumulation of the alternatively spliced and polyadenylated mRNAs are temporally regulated during the virus life cycle, producing distinct mRNA species at different time points during the infection [7,8,9].

Most of the adenoviral transcription units encode for two or more alternatively spliced mRNAs (Figure 1). Given the compact genome of adenovirus, regulatory events taking place at the level of RNA processing are of great importance for the lytic virus life cycle by controlling the synthesis of mRNAs that are translated to proteins that are needed at certain stages of the viral life cycle. The general tendency is that shorter mRNAs, produced by splicing out larger introns, accumulate at later time points of infection [2]. Further, viral introns typically do not interrupt the open reading frame of the gene (the noticeable exceptions are E1A, L4-33K and the U exon protein). It is also noteworthy that adenovirus genes contain few introns compared to cellular genes. Most viral mRNAs mature by removal of one to three introns. Since the virus has to compress much genetic information into a small DNA genome the selection appears to have favored few and short introns. A recent deep-sequencing work of Zhao* et al.* [9] reported an updated description of the adenovirus type 2 splicing profile that confirmed the kinetics of the viral transcripts expression and also identified a number of previously undocumented splice sites.

### 2.1. The Major Late Transcription Unit

The accumulation of mRNAs from the adenovirus major MLTU is subjected to a tight regulation at multiple levels, including transcription initiation, elongation, poly(A) site choice, pre-mRNA splicing, and translation (reviewed in [7,10]). The MLTU produces a primary transcript of approximately 28,000 nucleotides, which become polyadenylated at one of five positions, producing five families of mRNAs with co-terminal 3'-ends, the so-called L1 to L5 family of mRNAs (Figure 1). The MLTU transcripts use alternative splicing to produce approximately 20 different mRNAs. The major late promoter (MLP) is active both early and late after infection. However, at early times it produces shortened transcripts that gradually pre-terminate downstream of the L1 poly(A) site [11,12,13]. At this stage of infection, the only MLTU mRNA accumulation in the cytoplasm is the L1 52,55K mRNA. After the onset of the late phase, transcription is extended and mRNAs from L1 to L5 regions are produced [14,15,16]. Interestingly, at an intermediate phase of infection, after viral DNA replication has commenced but before the onset of viral late protein synthesis, the L4 family of mRNAs starts to accumulate [17]. More recent studies have suggested that the L4 mRNA expression at this time point is controlled by a novel L4 specific promoter [18,19].

#### 2.1.1. The L1 Model System

**Figure 2:**
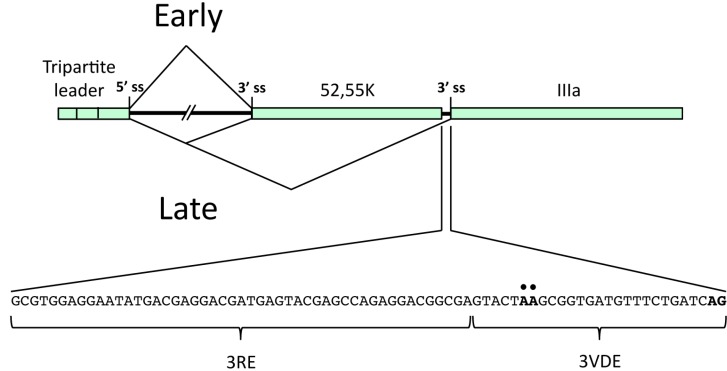
Schematic drawing of the regulation of Ad5 L1 alternative RNA splicing. The early and the late expression pattern are represented in the upper and lower part of the drawing respectively. In the inset, the sequences of the IIIa repressor element (3RE) and of the IIIa virus-infection dependent splicing enhancer (3VDE) are indicated. The black dots mark the IIIa branch sites and the AG in bold the IIIa 3' splice site.

L1 represent an example of an alternatively spliced gene where the last intron is spliced using a common 5' splice site and two competing 3' splice sites, generating two cytoplasmic mRNAs, the 52,55K and the IIIa mRNAs, respectively (Figure 2). Further, L1 is the only unit in the MLTU producing mRNAs both early and late during the virus life cycle. The pattern of L1 pre-mRNA splicing is subjected to a tight temporal regulation. Thus, within a 12 h time frame the specificity of the cellular RNA splicing machinery is remodeled from producing exclusively the 52,55K mRNA at early times of infection to producing predominantly the IIIa mRNA at the late phase of infection. The 52,55K protein has a role in viral genome encapsidation by stabilizing the association of the viral genome with the preformed empty capsids [20,21] whereas the IIIa protein serves its best characterized function as a structural protein in the capsid [22].

The L1 unit has been extensively used as a model transcript for studies of the temporal control of adenovirus alternative RNA splicing. The results have shown that IIIa splicing is subjected to a tight control by two *cis*-acting viral elements: the IIIa repressor element (3RE), and the IIIa virus-infection dependent splicing enhancer (3VDE) located in the intron just upstream of the IIIa 3' splice site (Figure 2).

The 3RE functions as a negative element in early virus-infected cells and block spliceosome assembly on the IIIa 3' splice site by binding the hyper-phosphorylated form of the cellular SR family of splicing factors [23,24,25]. This inhibition is relieved by the viral E4-ORF4 protein, which induces SR protein dephosphorylation [26,27]. E4-ORF4 is a multifunctional viral regulator that binds to the cellular protein phosphatase PP2A and blocks E1A-induced transcription activation [28,29,30], induces hypo-phosphorylation of various viral and cellular proteins [28,31] and regulates adenovirus alternative RNA splicing [26,27]. The E4-ORF4 induced SR protein dephosphorylation relieves the repressive function of SR proteins on IIIa splicing and makes the branch point accessible for U2 snRNP recruitment [26]. The primary SR protein target appears to be SRSF1 [27]. Since SR proteins are essential for generic pre-mRNA splicing, this virus-induced dephosphorylation may play a role in the inhibition of host cell gene expression at late times of infection.

The second element, the 3VDE, is the most critical element controlling IIIa pre-mRNA splicing. This element functions as a Janus element: it blocks splicing in HeLa-NE and activates splicing only in the context of an adenovirus infection [32]. Furthermore, this element is both necessary and sufficient to convert a cellular pre-mRNA, like β-globin, to a pre-mRNA that shows the same splicing phenotype as the, *bona fide*, viral IIIa pre-mRNA. The 3VDE consists of the IIIa branch site, the weak pyrimidine tract and the 3' splice site AG (Figure 2). Mutating the IIIa pyrimidine tract essentially abolishes IIIa 3' splice site activation. Interestingly, available results suggest that the IIIa 3' splice site activation in late adenovirus infected cells occurs without a stable binding of the general splicing factor U2AF to the IIIa pyrimidine tract [32,33,34]. Instead a hypothetical factor, the 3VDE interacting factor (3VDF), appears to replace the function of U2AF in U2 snRNP recruitment. As we will describe below, the adenoviral L4-33K protein appears to be the critical viral constituent of 3VDF.

The proliferation of the complex set of mRNAs expressed from the MLTU coincides with the activation of L4 mRNA expression at the intermediate time of infection [17]. In the early days this result was interpreted to indicate that the L4 unit might encode for proteins that are required for the shift from the early to late specific profile in mRNA expression, a prediction that current data strongly support. Here we summarize the recent results that have strengthened this prediction.

#### 2.1.2. The L4 Transcript Proteins

**Figure 3:**
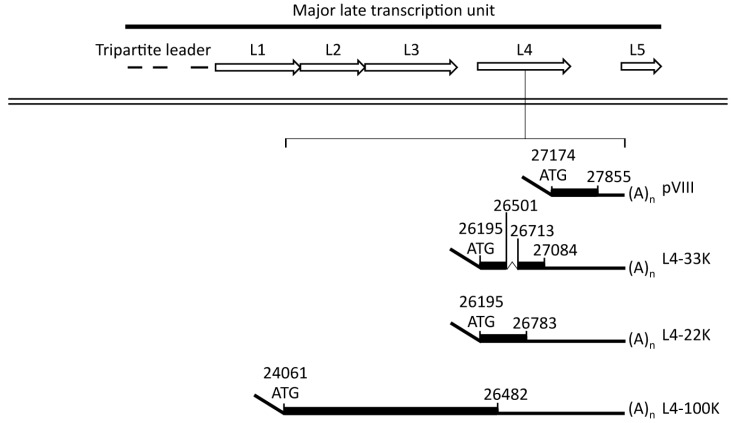
Schematic drawing highlighting the structure of the mRNAs expressed from the L4 unit. The adenoviral major late transcription unit (MLTU) contains five coding regions called L1-5, indicated by open arrows. The dsDNA adenoviral genome is represented as a double black line. The L4 transcripts (pVIII, L4-33K, L4-22K, L4-100K), with the corresponding genomic regions in Ad5 and their splicing pattern, are indicated.

The adenovirus genome is small and packed with information. This is exemplified by region L4, which is crowded with information and contain few surplus nucleotides. For example, the Ad5 L4 region encodes five mRNAs that are translated into four distinct proteins (L4-100K, L4-22K, L4-33K and pVIII) that serve various functions in the viral life cycle (Figure 3). The pVIII protein is a structural component of the virus capsid [35] whereas the L4-100K protein stimulates hexon trimer formation and nuclear import [36] and further promotes the selective translation of viral late mRNAs by stimulating the recruitment of the ribosomal 40S subunit to tripartite leader-containing viral mRNAs [37].

The L4-22K and L4-33K mRNAs encodes for two related proteins that share the first 105 amino acids but have unique *C*-terminal ends. L4-33K is translated from a spliced mRNA where an intron interrupting the protein coding sequence has been removed whereas L4-22K is translated from the corresponding unspliced mRNA (Figure 3). Further, the 95 amino-terminal amino acids in the L4-22K and L4-33K proteins overlap the carboxy-terminal end of the L4-100K protein in an alternate reading frame. L4-22K and L4-33K have been implicated as factors required for virion assembly [38,39] and appear to be key regulators in the switch from the early-to-late phase of infection. 

The L4 mRNAs are expressed from two different promoters dependent on the time of infection. At intermediate times of infection when L4 mRNA expression becomes selectively activated from the MLTU [17], a novel L4 specific promoter (L4P) (Figure 1), which is embedded in the L4-100K open reading frame, starts to function [19]. L4P is activated by the early and intermediate viral proteins E1A, E4-ORF3, IVa2, and the cellular p53 protein [19,40]. The p53 protein appears to be a critical factor inducing L4 mRNA expression and associates transiently to the L4P promoter to initiate L4 gene expression. Synthesis of the L4-22K and L4-33K proteins then establishes a negative feed-back loop that turns on the transient activity of the L4P and promotes an activation of the MLTU gene expression both by activating the MLP at the transcriptional level and MLTU gene expression at post-transcriptional levels [18,41,42,43]. Consequently, at late times of the infection the L4 mRNAs are transcribed as all the other MLTU mRNAs from the MLP [14].

### 2.2. L4-22K

**Figure 4:**
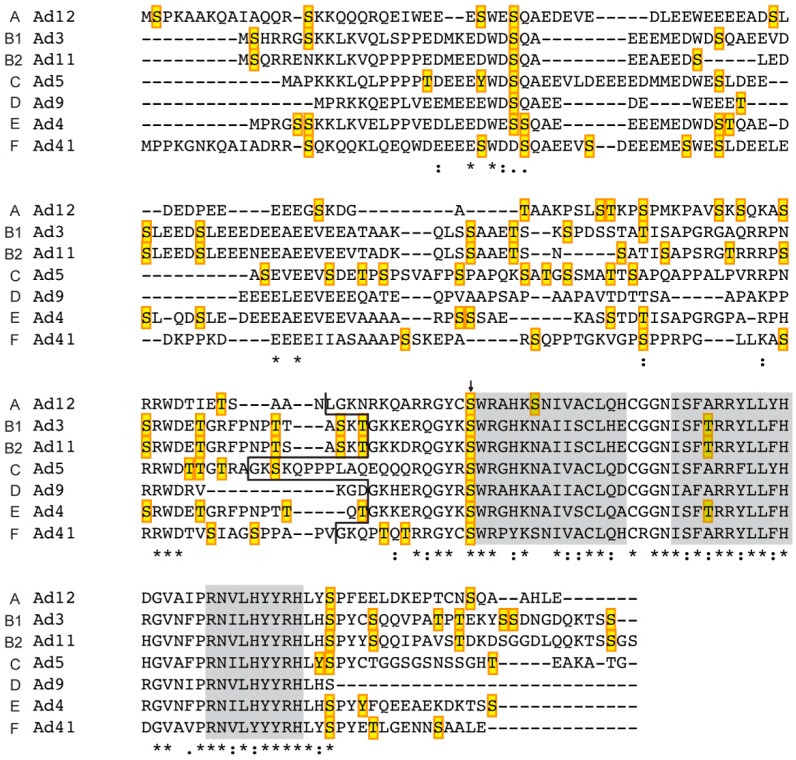
Alignment of L4-22K proteins from different human serotypes and their putative phosphorylation sites. The amino acid sequence of the indicated L4-22K proteins was aligned using the Clustal Omega software. The conserved residues are indicated by star (*), colon (:), and period (.) according to the level of identity and similarity of the amino acid aligned: full identity, amino acids with similar characteristics, and weakly similar residues, respectively. The black line separates the *N*-terminal part (shared with the respective L4-33K) and the *C*-terminal region of each serotype. The predicted helical domains identified by Predict Protein on the Ad5 L4-22K polypeptide sequence are shown in the grey boxes. The putative phosphorylation sites detected with NetPhosK 1.0 are marked in yellow boxes and the arrow indicates the conserved residues predicted to be phosphorylated by the same kinase (see also Table S1A).

The L4-22K protein serves two essential functions during a lytic infection. The protein is required for viral DNA encapsidation, by binding to a conserved TTTG motif in the A repeats in the packaging domain [38,44]. Also, the L4-22K protein is important for the early to late switch in viral gene expression by post-transcriptional regulation of mRNA production/stability [18,45]. Interestingly, these two functions can be separated. Thus, a mutation of the conserved cysteines (C137, C141) resulted in a mutant virus with a defect in the formation of infectious viral particles but showed no impairment in the capacity to regulate viral gene expression [46]. In contrast, mutating the conserved histidines (H166, H170) resulted in a mutant that showed a phenotype that approached the defects observed with a virus completely devoid of L4-22K protein expression. Surprisingly, despite these phenotypic defects the cysteine- or histidine-mutated L4-22K proteins were still able to bind to the A repeats in the packaging sequence [46], suggesting that the potential cysteine histidine Zn-finger does not function as the much looked for DNA binding domain. Interestingly, the analysis of Ad5 L4-22K amino acidic sequence by PredictProtein [47] suggested the presence of three helical domains in the *C*-terminus of the protein (aa 127–140, 145–155, 162–170). The alignment of the L4-22K protein sequence from members of the different subgroups (A, B1, B2, C, D, E, F) performed with Clustal Omega software [48] suggests that the unique *C*-terminus contain two helical domains that are highly conserved (Figure 4).

Late-specific activation of the MLP is dependent on elements located downstream of the transcriptional start site, the so-called R1 region and the DE element [49]. The DE element binds two virus-induced transcription factors, known as DEF-A and DEF-B [50,51,52]. The core factor of DEF-A appears to be the L4-22K protein [41] whereas DEF-B seems to be a homodimer of the adenoviral IVa2 protein [42,50] The L4-22K protein binds to the DE sequence and activate MLP transcription both in cell culture experiments and* in vitro*, while the IVa2 protein does not activate MLP transcription by itself and only marginally stimulates L4-22K activation of transcription in transient transfection experiments [41]. However, available data suggests that the IVa2 protein may selectively enhance MLP transcription during a virus infection (reviewed in [11]). Previous experiments have also demonstrated that both the IVa2 and the L4-22K proteins bind to the viral DNA packaging domain, which consists of seven related elements, called A repeats (reviewed in [53]). However, it should be noted that it has been reported that the L4-33K is the L4 protein binding to the DE element as well as to the core sequence of the viral packaging domain [54]. Still, more work is required to sort out the exact contribution of the two L4 proteins in the various processes. From this point, it might be worth reminding that L4-22K and L4-33K share a large part of their amino acid sequence through the identical amino-terminal end (Figure 3).

Several reports have suggested a possible role of the L4-22K protein also in RNA processing and/or additional gene regulatory processes. In the context of a virus infection L4-22K stimulates the accumulation of only a selected set of late genes [18]. For example, the stimulatory effect of L4-22K on protein V and penton base expression was manifested at the level of an increase in spliced V and penton base mRNA accumulation, suggesting an effect on splicing/stability/nuclear export. In contrast, L4-22K stimulated hexon protein accumulation without enhancing hexon mRNA abundance, suggesting an effect on translation and/or protein stability [18]. Also, L4-22K is required for the late expression of the adenovirus death protein (ADP) encoded by the E3 region [45]. However, at late times of infection E3 ADP is expressed as part of the MLTU [55]. Further, the L4-22K protein has also been shown to directly control accumulation of the L4-33K mRNA, at the level of RNA splicing [46]. 

Interestingly, L4-22K was also demonstrated to affect the expression of some early genes. In an* in vivo* model based on mutant viruses encoding truncated L4-22K proteins, the expression of the E1A and the E2-72KDBP increased, while intermediate and late protein expression was decreased or delayed [45]. Previous data from our group has shown that the E1A promoter, which contains the A-repeats of the DNA packaging signal is not activated by L4-22K in an* in vitro* transcription assay [41]. Taken together, these observations suggest that L4-22K might play a role in the regulation of the viral gene expression in a complex way and potentially at multiple post-transcriptional levels. 

### 2.3. L4-33K

Like L4-22K, the L4-33K protein has also been demonstrated to play a key role in the early-to-late switch in adenovirus gene expression by promoting the cytoplasmic accumulation of late mRNAs [56,57] and, at least* in vitro*, activate splicing of MLTU pre-mRNAs with a weak 3' splice site context [43]. Several previous reports also indicated that L4-33K might function as a virus assembly factor [39,58,59,60]. Thus, by introducing stop codons in the unique *C*-terminal part of the protein, the production of infectious virus particles was shown to fail completely [59]. A recent finding corroborates these findings and show that an L4-33K mutant virus produces empty viral capsids and is impaired in viral genome packaging [57]. The results further suggested that L4-33K itself does not bind to the viral packaging sequence and that the mutant virus defective in L4-33K expression did not show any defects in the binding capacity of other well-characterized viral packaging proteins, like L4-22K [57]. In contrast, other studies have suggested that L4-33K associates to the packaging domain, even though it was not clear whether the binding was direct or indirect [39,54]. These reports exemplify the current complex nature of L4-33K function(s).

Even though L4-33K has been suggested to function as a transcription factor stimulating the MLP [54], several reports suggest that a primary function of the protein is as a splicing factor activating L1 alternative splicing [41,43,57,61]. In fact, L4-33K appears to be the key regulator of L1 alternative splicing since expression of this viral protein alone in HeLa cells is sufficient to shift the L1 alternative splicing pattern from the early to the late-specific pattern observed in an adenovirus late-infected cell [43].

**Figure 5:**
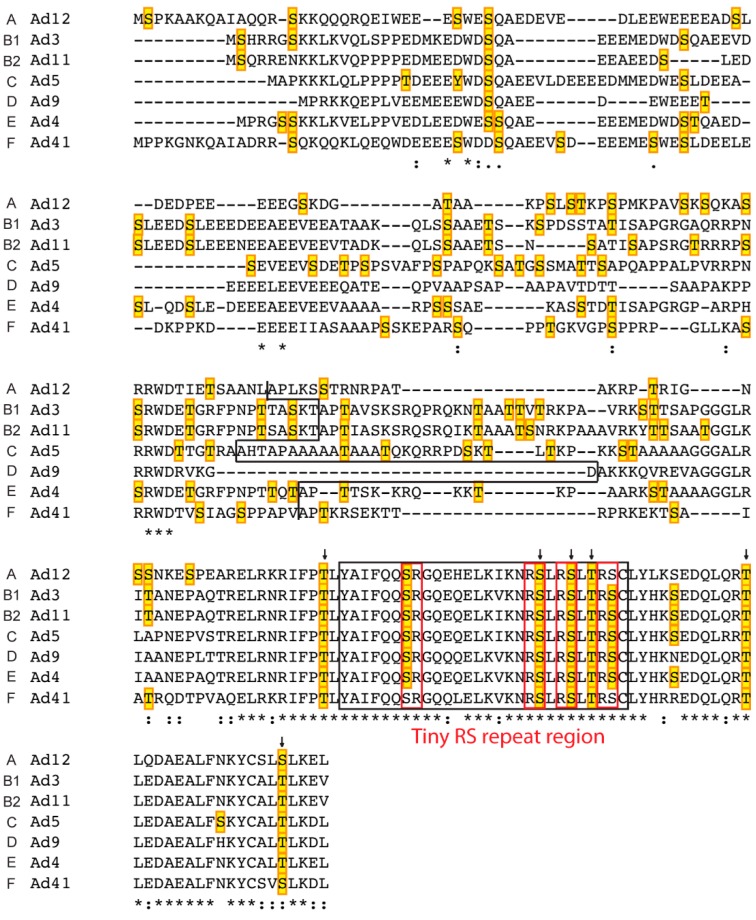
Alignment of L4-33K proteins from different human serotypes and their putative phosphorylation sites. The amino acid sequence of the indicated L4-33K proteins was aligned using Clustal Omega software. The conserved residues are indicated by star (*), colon (:), and period (.) according to the level of identity and similarity of the amino acid aligned: full identity, amino acids with similar characteristics, and weakly similar residues, respectively. The black line separates the *N*-terminus (shared with the respective L4-22K) and the *C*-terminal region of each serotype. The putative phosphorylation sites detected with NetPhosK 1.0 are marked in yellow boxes, and the conserved residues predicted to be phosphorylated by the same kinase are indicated by arrows (see also Table S1B). The ds domain is shown with a black-box with the tiny SR repeats indicated in red boxes.

L4-33K activates L1-IIIa splicing primarily through the 3VDE (Figure 2) making this protein the leading candidate for being the still uncharacterized 3VDF, or at least the viral component of the 3VDF. In agreement with this, L4-33K preferentially activates transcripts with weak 3' splice sites, the sequence context typical for many of the 3' splice sites in the MLTU that are activated at the late stage of infection [43]. Surprisingly, other studies have suggested that in the context of a virus infection L4-33K is not the global regulator of MLTU alternative RNA splicing and shows a substrate specificity limited to the activation of IIIa and pVI alternative pre-mRNA splicing [57]. Since the L4-22K protein also has been shown to regulate accumulation of the major late mRNAs the two proteins may serve an as yet unexplained redundant function in the control of gene expression. At this point it is worth mentioning that in Ad5 the proteins share the 105 *N*-terminal amino acids (Figure 4 and Figure 5). No function has yet been assigned to this part of the protein but future studies might shed new light on a potential contribution to virus replication.

The unique *C*-terminus of L4-33K functions as the L1-IIIa splicing enhancer domain* in vitro* and* in vivo* [43] and is highly conserved between different human serotypes (Figure 5). The splicing domain contains a tiny RS repeat, making the protein slightly resemble the SR family of splicing factors [62,63]. However, the RS repeat in L4-33K is much too short [64] to classify L4-33K as a viral SR protein. Substituting serines to glycines in the tiny RS repeat suggested that serine192 is essential for the L4-33K splicing enhancer function, whereas three other serines in the tiny RS repeat served redundant functions [43,61]. The tiny RS repeat region is contained within the so-called double-splice (ds) domain [43]. The ds domain corresponds to the *C*-terminal amino acids 170–197 of the L4-33K protein, which are taken out by a double-splicing event of the L4-33K reading frame [43].

L4-33K localizes to the nuclear margin in cells transiently transfected with a plasmid expressing L4-33K [61]. Interestingly, infection of the transfected cells resulted in a relocalization of the L4-33K protein to the periphery of the viral replication centers. Deletion mutants lacking the tiny RS repeat or having Ser^192^ mutated were defective in nuclear localization and failed to redistribute to the viral replication centers. Interestingly, in the bovine adenovirus 3 L4-33K protein the tiny RS region has been shown to be required for interaction with the nuclear import receptor, transportin-3 [65], which is implicated in the nuclear import of splicing factors including SRSF1 (ASF/SF2) [66].

## 3. Post-Translational Modification of Adenoviral Proteins

Viral proteins are frequently subjected to post-translational modifications that might affect their function [67]. Several experimentally validated phosphorylations have been reported for the non-structural proteins of the closely related Ad2 and Ad5: for example, E1A [68,69,70,71,72,73,74], E1B [75,76], E2A-72KDBP [77], L4-100K and L4-33K [78,79], E3 RID-beta [80], E4-ORF4 [81] and Ad-Pol [82,83]. By mass spectrometry of purified virus particles it was recently also demonstrated that several of the structural proteins of Ad2 are extensively phosphorylated [84,85].

### 3.1. A Janus Effect of Two Cellular Protein Kinases on the Activity of L4-33K as an Alternative Splicing Enhancer Protein

In the early studies three viral proteins (L4-100K, L4-33K and E2A-72K-DBP) were found to be the major phospho-proteins expressed during an adenovirus infection [78,79]. The L4-33K and L4-22K proteins share the 105 *N*-terminal amino acids (Figure 4 and Figure 5). This part of the proteins is not conserved between L4 proteins from different serotypes. However, they contain a high content of acidic amino acids, a feature that appears to be the conserved feature among the L4 proteins from human adenoviruses.

L4-33K has been characterized as a viral alternative RNA splicing factor activating L1 IIIa pre-mRNA splicing and other 3'-splice sites with a weak sequence context [43]. Two cellular protein kinases, DNA-PK and PKA, have been shown to phosphorylate the unique *C*-terminal domain of the Ad5 L4-33K protein, resulting in opposite outcome on L1 alternative splicing [86]. The L4-33K protein specifically associates with the catalytic subunit of DNA-PK (DNA-PKcs) both in pull down assays and during a lytic adenovirus infection. DNA-PK is a heterotrimeric enzyme composed of the DNA-PKcs and two regulatory subunits, Ku86 and Ku70, which facilitate the recruitment of DNA-PKcs to DNA double stranded breaks. In fact, phosphorylation of most DNA-PK substrates is activated by linear DNA. However, the L4-33K protein does not bind to the regulatory Ku subunits, a finding that is in agreement with the observation that DNA-PK phosphorylation of L4-33K is double stranded DNA-independent [86]. Using two cell lines that differ in their expression of DNA-PKcs it was demonstrated that DNA-PK had an inhibitory effect on the early to late switch in L1 IIIa 3' splice site activation [86]. These results suggested that DNA-PK might be an important factor controlling adenovirus MLTU alternative splicing during a lytic infection.

Interestingly, DNA-PK is targeted by three additional adenoviral proteins, the E4-ORF3, E4-ORF4 and E4-ORF6 proteins [31,87,88]. Since DNA-PK is an essential factor involved in the double strand DNA repair pathway [89], the E4-ORF3 and E4-ORF6 proteins serve an important function to prevent concatemerization of the linear viral DNA genome during a lytic infection [88,90,91]. From this perspective it is interesting to note that E4-ORF3 and E4-ORF6 also have been shown to function as alternative RNA splicing factors both in transient transfection assays and during a lytic infection [92,93]; the E4-ORF3 protein stimulates tripartite leader splicing by facilitating i-leader exon inclusion, whereas the E4-ORF6 protein favors i-leader exon skipping. The significance of E4-ORF4 binding to DNA-PK was not investigated in Lu, *et al.* [31]. However, as described above, E4-ORF4 is a regulator of alternative RNA splicing by causing SR protein dephosphorylation [26,27]. It needs to be tested whether the four proteins target DNA-PK in a temporal manner during the virus infectious cycle.

L4-33K is also an excellent substrate for PKA phosphorylation* in vitro* [86]. However, a stable interaction between the kinase and the substrate could not be established suggesting that the interaction may be indirect or alternatively occur via a hit and run mechanism. Interestingly, both protein kinases phosphorylate L4-33K within the unique *C*-terminal splicing enhancer domain [86], in agreement with the observation that both protein kinases have a regulatory effect on L1 alternative splicing. In the case of DNA-PK the predominant phosphorylation was further shown to reside within a short peptide containing the tiny RS repeat [86], which has been shown to be essential for the function of L4-33K as an alternative splicing factor [43]. Interestingly, the tiny RS repeat is not phosphorylated by the classical SR protein kinases SRPK1 or Clk/Sty [43], which previously have been shown to be key regulators of SR protein function [94].

Undoubtedly, the most interesting finding in the Törmänen study [86] was that the two kinases have a regulatory and opposite effect on the early to late shift in L1 alternative RNA splicing. Thus, DNA-PK functioned as an inhibitor of the shift in L1-52,55K to L1-IIIa 3' splice site usage whereas PKA activated distal L1-IIIa mRNA splicing. However, details how the protein kinases affected L4-33K function was not provided. Clearly, much more work will be required to clarify the target specificity and the mechanism(s) by which L4-33K control adenovirus MLTU alternative RNA splicing.

### 3.2. Prediction of Phosphorylation Sites in L4-33K and L4-22K

Since the amino-terminus is shared between the L4-33K and L4-22K proteins from the different adenovirus serotypes, both proteins may be subjected to a similar regulation by cellular protein kinases. With the aim of identifying the potential post-translational phosphorylation sites that might affect the function(s) of the L4-22K and L4-33K proteins of different serotypes (Ad12, Ad3, Ad11, Ad5, Ad9, Ad4, Ad41) we performed a prediction analysis of the respective amino acidic sequences by using the NetPhosK 1.0 [95] software. This analysis was integrated with the alignment results obtained by the Clustal Omega program [48], which allowed us to identify the pattern of conservation between the L4 proteins of different adenovirus serotypes (Figure 4 and Figure 5). The majority of the potential phosphorylation sites are localized in the N-terminal part of the proteins, suggesting that L4-22K and L4-33K may be subjected to a similar type of regulation. 

The putative phosphorylation sites found throughout the L4-22K sequence of the different serotypes, accounted for 10%–12% of the total number of residues, with the exception of Ad9 L4-22K, whose predicted sites were 2% of the residues (Table S2A). Also the Ad9 L4-33K protein appears to be phosphorylated to a lower extent (6%) compared to the L4-33K proteins from other serotypes (11%–15%) (Table S2B). All the L4-22K proteins analyzed were found to be potential substrates for ATM, CKI, CKII, DNAPK, and PKC phosphorylation (Table S2A). Further, a conserved serine residue located just upstream of the first predicted helix in Ad5 L4-22K protein was found to be a putative target of PKC phosphorylation for all the serotypes (Figure 4 and Table S1A). The L4-33K proteins are potential substrates for cdc2, CKI, CKII, DNAPK, PKA, and PKC phosphorylation (Table S2B). Additionally, the candidate phospho-sites in the conserved region of the L4-33K proteins are putative targets of the same kinases (Table S1B), suggesting a potential for a shared mechanism to regulate L4-33K protein activity among the different adenovirus families. Three conserved threonine residues localized outside the ds domain (T168, T209, T224 in Ad5 L4-33K) are potential phosphorylation sites for PKA, cdc2, PKC and PKA respectively (Figure 5 and Table S1B). In the ds domain, crucial for the function of L4-33K as a splicing enhancer factor, two conserved serines and a threonine (S189, S192, T194 in Ad5 L4-33K) are predicted to be phosphorylated by PKA and PKC (Figure 5 and Table S1B).

Further analyses were performed on the Ad5 proteins by using different informatics tools like scan-x [67] and PredictProtein Prosite [47]. The analysis on Ad5 only proteins performed with scan-x [67] suggested Y19, S22, S41, S47, S53, and S65 as potential phosphorylation sites, thus confirming part of the sites found by the NetPhosK 1.0 analysis. In addition, an extra site was predicted on S180 of Ad5 L4-22K. The conserved serine 126 upstream of the first predicted helix in L4-22K (Figure 4, and see above) was found to be a putative target of PKC phosphorylation by both Prosite and NetPhosK 1.0. In* in vitro* phosphorylation assays, L4-22K was a poor substrate for PKA phosphorylation [86], in agreement with the prediction that only one site (T100) is a potential PKA phosphorylation site (Table S2A). On the other hand, L4-22K was phosphorylated by DNA-PK, although to a much lesser extent than L4-33K [86]. Collectively, these predictions of conserved post-translational modification sites are in line with the hypothesis that the function of L4-22K as a virus assembly factor and a regulator of gene expression might be affected by post-translational modifications induced by cellular protein kinase(s).

**Figure 6:**
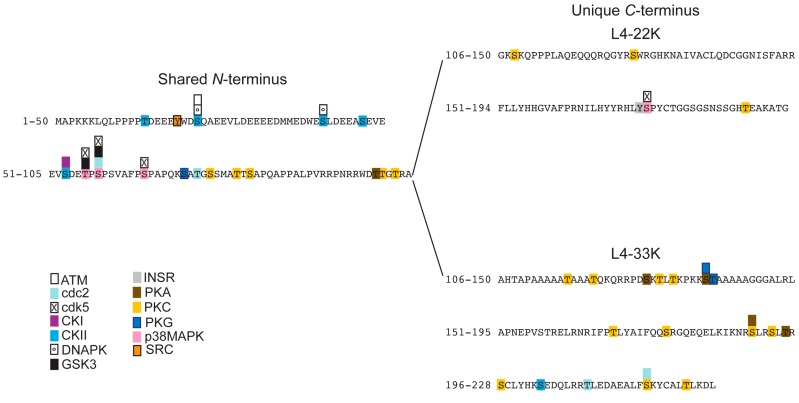
Predicted phosphorylation sites of Ad5 L4-33K and L4-22K proteins. The shared *N*-terminal region is shown on the left, and the unique L4-22K and L4-33K *C*-termini are on the right. The result of the phosphorylation sites prediction performed with NetPhosK 1.0 is graphically shown as boxes for the respective kinases in the corresponding predicted residue, according to the color code in the figure. Amino acids potentially phosphorylated by more kinases are marked by piled boxes, according to the kinases and the respective score (lowest score on top and highest on the protein sequence).

L4-33K is a major phospho-protein expressed during an adenovirus infection [78,79]. The analysis of the amino acidic sequence of Ad5 L4-33K revealed the presence of several potential phosphorylation sites (33 serines/threonines/tyrosines) throughout the protein (Table S2B and Figure 6). Even though half of these are present in the common part shared with Ad5 L4-22K, the unique* C*-terminal region in L4-33K is predicted to be a better substrate for phosphorylation compared to the *C*-terminus of L4-22K (Figure 6).

The Ad5 L4-33K protein is an excellent substrate for DNA-PK phosphorylation* in vitro* [86]. The primary DNA-PK phosphorylation sites have been mapped by this* in vitro* assay to the unique *C*-terminal part of L4-33K (Figure 6). More specifically the ds domain (Figure 5) appears to contain the main DNA-PK phosphorylation sites in Ad5 L4-33K. Using the NetPhosK 1.0 software prediction tool DNA-PK is predicted to phosphorylate the Ad5 L4-33K at two sites in the *N*-terminal part of the protein (S22 and S41). PredictProtein analysis identified further residues of Ad5 L4-33K potentially subjected to phosphorylation by PKC (S157) and CKII (S157, T209, T224), besides others also identified with NetPhosK 1.0 (S137 for PKA; T120, S189, and T224 for PKC; T14, S22, S41, S47 for CKII).

Clearly, an understanding of the complex and balanced phosphorylation of these two L4 products will require much work to determine the phosphorylated status of the proteins during an infection, and more importantly a mutant analysis to establish the functional consequence of these phosphorylation events. Additionally, considering the different tropism, the differences in immune response, and the different outcome of an infection (lytic/persistent) might further complicate the interpretation of the contribution of the two L4 proteins for different adenovirus infections [6,8,35,96,97].

## Supplementary Materials

Supplementary materials can be found at http://www.mdpi.com/1422-0067/16/02/2893/s1.
